# Risk factors for antepartum stillbirth: a case-control study in Nepal

**DOI:** 10.1186/s12884-015-0567-3

**Published:** 2015-07-05

**Authors:** Ashish KC, Viktoria Nelin, Johan Wrammert, Uwe Ewald, Ravi Vitrakoti, Geha Nath Baral, Mats Målqvist

**Affiliations:** Department of Women’s and Children’s Health, International Maternal and Child Health, Uppsala University, SE-751 85 Uppsala, Sweden; United Nation’s Children’s Fund, Nepal Country Office, UN House, Pulchowk, Nepal; Foundation for Maternal and Child Health Nepal, Kathmandu, Nepal; Paropakar Maternity and Women’s Hospital, Kathmandu, Nepal

**Keywords:** Antepartum stillbirth, Risk factors, Nepal

## Abstract

**Background:**

Globally, at least 2.65 million stillbirths occur every year, of which more than half are during the antepartum period. The proportion of intrapartum stillbirths has substantially declined with improved obstetric care; however, the number of antepartum stillbirths has not decreased as greatly. Attempts to lower this number may be hampered by an incomplete understanding of the risk factors leading to the majority of antepartum stillbirths. We conducted this study in a tertiary hospital in Nepal to identify the specific risk factors that are associated with antepartum stillbirth in this setting.

**Methods:**

This case-control study was conducted between July 2012 and September 2013. All women who had antepartum stillbirths during this period were included as cases, while 20 % of all women delivering at the hospital were randomly selected and included as referents. Information on potential risk factors was taken from medical records and interviews with the women. Logistic regression analysis was completed to determine the association between those risk factors and antepartum stillbirth.

**Results:**

During the study period, 4567 women who delivered at the hospital were enrolled as referents, of which 62 had antepartum stillbirths and were re-categorized into the case population. In total, there were 307 antepartum stillbirths. An association was found between the following risk factors and antepartum stillbirth: increasing maternal age (aOR 1.0, 95 % CI 1.0–1.1), less than five years of maternal education (aOR 2.4, 95 % CI 1.7–3.2), increasing parity (aOR 1.2, 95 % CI 1.0–1.3), previous stillbirth (aOR 2.6, 95 % CI 1.6–4.4), no antenatal care attendance (aOR 4.2, 95 % CI 3.2–5.4), belonging to the poorest family (aOR 1.3, 95 % CI 1.0–1.8), antepartum hemorrhage (aOR 3.7, 95 % CI 2.4–5.7), maternal hypertensive disorder during pregnancy (aOR 2.1, 95 % CI 1.5–3.1), and small weight-for-gestational age babies (aOR 1.5, 95 % CI 1.2–2.0).

**Conclusion:**

Lack of antenatal care attendance, which had the strongest association with antepartum stillbirth, is a potentially modifiable risk factor, in that increasing the access to and availability of these services can be targeted. Antenatal care attendance provides an opportunity to screen for other potential risk factors for antepartum stillbirth, as well as to provide counseling to women, and thus, helps to ensure a successful pregnancy outcome.

**Clinical trial registration:**

ISRCTN97846009 (url. www.isrctn.com/ISRCTN97846009)

## Background

Globally, at least 2.65 million stillbirths (birth weight ≥1000 g or ≥28 weeks of gestation) occur every year; and of these, more than half (1.45 million) occur during the antepartum period [1]. Among the total number of antepartum stillbirths that took place globally in 2009, 470,000 (32 %) took place in South Asia [1, 2]. In high-income countries, the proportion of stillbirths occurring during the intrapartum period has substantially declined with improved obstetric care; conversely, the number of antepartum stillbirths has not decreased as greatly [3]. This indicates that even in high-income countries, strategies based on the identification of high-risk pregnancies have not been successful in preventing antepartum stillbirth.

Attempts to lower the stillbirth rate further may be hampered by an incomplete understanding of the risk factors leading to the majority of antepartum stillbirths. There are several risk factors which have been associated with antepartum stillbirth in studies from high-income countries; maternal age greater than 35 years, parity higher than four, low maternal educational status, lack of antenatal care attendance, chronic maternal medical conditions, pre-eclampsia or placenta abruption during pregnancy, intra-uterine growth restriction, major congenital anomaly of the infant, and poor maternal nutritional status [4–6]. Additionally, a prospective cohort study from the Netherlands found that substandard clinical care during pregnancy was a risk factor for antepartum stillbirth among term infants [7]. A population-based cohort study in rural Ghana (a lower-middle-income country) found an association of antepartum stillbirth with previous stillbirth, increasing maternal age (>35 years), primiparity, multiple pregnancies and no antenatal care attendance [8].

Prevention and reduction of antepartum stillbirth is especially important with the recent endorsement of the Global Every Newborn Action Plan by the 67^th^ World Health Assembly, which sets the global target to reach a stillbirth rate of less than 10 per thousand births by 2035 [9]. In 2011, Nepal had an estimated stillbirth rate of 22.4 per thousand births, with 80 % of these deaths occurring during the antepartum period [10, 11]. There has been a large reduction in the number of intrapartum stillbirth in the last 15 years in Nepal, however, the number of antepartum stillbirths has not declined as substantially [2]. Therefore, to reduce the current national stillbirth rate in order to reach the global target by 2035, reduction of antepartum stillbirth will be critical. Given the socio-economic and health situation in Nepal, there might be different structural and health related risk factors influencing the stillbirth rate than in high income countries, so it is important to assess the risk factors for antepartum stillbirth.

To the best of our knowledge, no studies have been conducted in South Asia or Nepal to identify risk factors for antepartum stillbirth, such that preventive and management strategies could be developed to reduce these preventable deaths. Therefore, we conducted this case-control study at a tertiary hospital in Nepal to identify the specific risk factors that are associated with antepartum stillbirth in this setting.

## Methods

### Study design

This study had an unmatched case-control design, nested within a larger hospital-based study aiming to evaluate the impact of a simplified neonatal resuscitation protocol on perinatal outcomes [12]. For the larger study purpose, a reference population was created to assess the change in the perinatal outcomes over a period of time. The reference population for the larger study was 20 % of the randomly selected women delivering in the hospital. For this study purpose, all the live birth from the reference population was selected as reference population and all antepartum stillbirth occurring during the study period was selected as case population. The sample size for the study was based on the larger study to detect the 20 % reduction in perinatal mortality with statistical power of 80 % and level of significance at 5 % [12].

### Setting

The study was conducted in a government-funded, tertiary hospital located in Kathmandu, Nepal. The hospital provides a range of obstetric and gynecological services and is staffed by 400 people. Each year, about 22,000 deliveries take place in the hospital through three delivery outlets; namely, the Maternal and Newborn Service Center for low-risk deliveries, the Labor Room for high-risk deliveries, and the operation theatre for high-risk and operative deliveries (Table 1). In 2011/12, the hospital had a stillbirth rate of 19 per 1000 births, with an estimated 220 stillbirths occurring annually [13].

**Table 1:** Human resources and set-up of each of the delivery units at the hospital

Delivery units	Type of Health workers	Number of HW	Number of delivery beds	Type of delivery service
Maternal and Newborn Service Center	Nurse midwives	11	8	Low-risk delivery
Labor Room	Obstetricians, medical doctors, nurse midwives	11	9	Low- and high-risk delivery
Operation room	Anesthesiologist, obstetricians, medical doctors, nurse midwives	11	1	Cesarean section

The hospital has a set clinical protocol for the initial assessment of women admitted for delivery. This protocol includes assessment of pre-pregnancy history, maternal medical conditions, and obstetric complications during pregnancy, and antenatal care attendance during the current pregnancy. A clinical examination is also done to determine gestational age, fetal status, stage of labor and fetal heart rate, using intermittent auscultation.

The study was completed between July 1, 2012 and September 30, 2013. As a part of the larger study evaluating the impact of neonatal resuscitation protocol implementation, ethical approval was obtained from the Institutional Review Committee of the Nepal Health Research Council (reg. No 37/2012) and Uppsala University, Sweden (dnr. 2012/267). The study was registered as clinical trial with the registration number: ISRCTN 97846009. Written consent was obtained from the women who volunteered to participate in the study.

### Participants

All women with antepartum stillbirth occurring during the study period were included as cases. Randomly selected referent women with live births or intrapartum stillbirths were included as referents. Any antepartum stillbirth occurring in the referent population was excluded from this group, re-categorized and included in the case population.

### Data collection

**Fig. 1:**
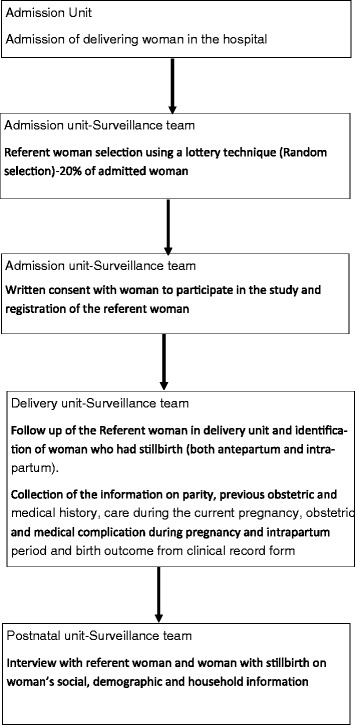
Data collection flow chart

For data collection, a surveillance team was set up under the guidance of a research manager (RV). There were 12 surveillance officers placed full time at the admission, delivery and postnatal units for data collection. Any woman admitted to the hospital for delivery was marked in the surveillance registry. From this sampling frame, study participants were randomly selected using a lottery technique. If a woman was selected as part of the referent population, she was tracked from the point of admission until discharge to assess labor progress and birth outcomes. Additionally, the surveillance officers tracked all women who had stillbirths occurring in the hospital. From both the referent and case populations, information on parity, previous obstetric and medical history, care during the current pregnancy, obstetric and/or medical complications during pregnancy, and intrapartum care was retrieved from clinical record forms. The surveillance team conducted structured interviews with each woman at their time of discharge using a questionnaire to evaluate social, demographic and household information (Fig. 1).

After the completion of the clinical record and interview forms for each woman, the research manager reviewed the forms for completeness. The data entry officer further reviewed and indexed each form to prevent data loss, as well as to ensure data security. Data was entered into a database for data cleaning using the Census and Survey Processing System (CS Pro) software (US Census Bureau and ICF International). This dataset was then exported to the Statistical Package for Social Sciences (SPSS) software (IBM Corporation) to complete data analysis.

### Variables

#### Antepartum stillbirth

Delivery of any non-viable fetus after 22 weeks of gestation, or with a birth weight more than 500 g, with an Apgar score of 0 at 1 and 5 min and signs of maceration, or absent fetal heart sound before the initiation of labor [1].

#### Parity

Number of times a woman gave birth after the age of viability, i.e. 22 weeks, including both live and still births.

#### Caste/Ethnicity

The caste to which a woman and her family belong, based on the social hierarchical system of caste that exists in Nepal [14].

#### Wealth index

The wealth index is a measure of socioeconomic position, used in national representative health surveys (Demographic Health Surveys) to compare socioeconomic inequalities [15, 16]. During the interviews with mothers, data were collected on ownership of durable assets (e.g. car, refigerator, bicycle, radio, television), housing characteristics (e.g. number of rooms, dwelling floor and roof materials, toilet facilities), and access to services (e.g. electricity supply, drinking water source). Using the scores from first principal component analysis, a wealth index (asset index) was contructed. Based on the value of this index, individuals were sorted and population quintiles were established using cut-off values. These quintiles were then ranked from bottom to top as poorest, poorer, middle, richer and richest [17].

#### Antenatal care attendance

Whether a mother attended any antenatal care visits, during which she received clinical examination, counseling and medication (if needed) from a health worker.

#### Antepartum hemorrhage

Vaginal bleeding before the onset of labor.

#### Hypertensive disorder of pregnancy

Maternal diastolic blood pressure of 90 mmHg or more in two consecutive assessments, which are at least four hours apart, during pregnancy.

#### Medical complication during pregnancy

Women having diabetes mellitus, severe anemia (Hb <7 gm/L), epilepsy, etc. during pregnancy.

#### Multiple pregnancy

Woman pregnant with more than one fetus.

#### Gestational age of the infant

Gestational age measurement was done based on the mother’s last menstrual period.

#### Birth weight

Weight of the baby was measured within 1 h of delivery using an analog pan scale.

#### Small-for-gestational age

Babies with a birth weight below the tenth percentile for a given gestational age and sex, based on a standard optimal reference population.

#### Appropriate-for-gestational age

Babies with a birth weight above the tenth percentile.

Since Nepal did not have a nationally representative population reference for birth weight according to gestational age and sex, we used the Alexander reference. This standard reference population included measurements from 3,134,879 nationally representative, multi-ethnic infants in the USA in 1991 [18].

The outcome variables, as well as exposure variables on parity, antenatal care attendance, and complications were assessed using each woman’s clinical record form.

The information on ethnicity, educational status, and wealth quintile was evaluated through semi-structured interview.

### Data analysis

For data analysis purposes, categorical variables were created from raw or continuous variables within the dataset. Maternal age was analyzed both as a continuous variable and as a categorical variable, grouping women into four groups as follows: <20, 20–25, 26–30 or 30 years of age and higher. A binary variable was created to categorize maternal education as primary school education (5 years) and less, or at least six years of schooling and above. Maternal ethnicity was categorized into six groups, as Brahmin/Chettri from the hill or terai region; relatively advantaged Janajatis, like Newar, Gurung and Thakali; disadvantaged Janajatis; non-Dalit from the terai region; Dalit from the hill or terai region; or Muslim. A wealth index consisting of five quintile groups was created, including poorest, poorer, middle, richer and richest quintiles. A binary variable was then created to categorize women as poor, i.e. those belonging to the poorest quintile, and non-poor, those belonging to any of the other four quintiles. Antenatal care attendance was categorized as having attended at least one visit or none. Parity was categorized into three groups including primiparous, multiparous (1–2) or multiparous (3 or more). The presence of previous stillbirth, antepartum hemorrhage during pregnancy, hypertensive disorder during pregnancy, any medical complication during pregnancy, multiple birth, or small weight-for-gestational age were categorized into binary variables as yes or no. And finally, the sex of the newborn was categorized as male or female.

Comparison of the demographic, social and obstetric characteristics among case and referent populations was done using Pearson’s chi-square and fisher’s exact test. A comparison of the mean and median maternal age in the two populations was done using a *t*-test.

For those demographic, social and obstetric characteristics that differed (*p* < 0.01) between the two population groups, univariate logistic regression analysis was conducted to test the association between those variables and antepartum stillbirth.

For those variables, which showed an association with antepartum stillbirth in the univariate logistic regression analysis a multivariate model was created to determine whether the association between the potential risk factors and antepartum stillbirth remained after adjusting for confounders. The variables investigated were maternal age (continuous), maternal education, wealth index (poor or non-poor), antenatal care attendance, parity, previous stillbirth, antepartum hemorrhage in pregnancy, hypertensive disorder during pregnancy, and small weight-for-gestational age.

We used the multiple imputation method to deal with data missing at random from the case or referent populations within the demographic, social, and/or obstetric variables [19].

## Results

**Fig. 2:**
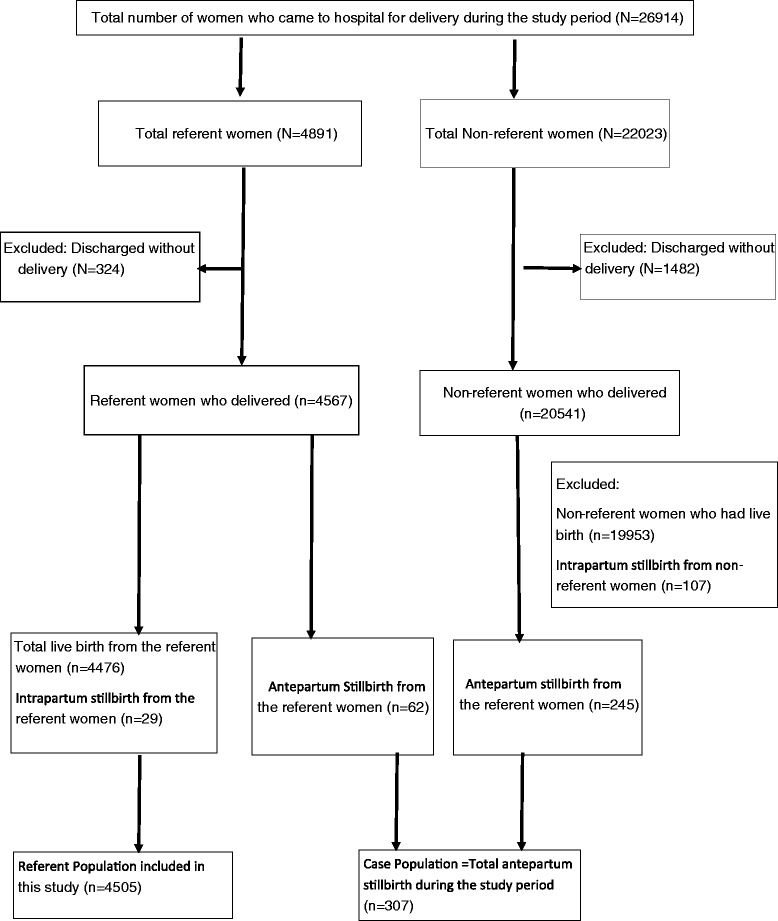
Case-referent study population

During the fifteen months of the study period, 26,914 women were admitted in the hospital for delivery. A total of 4567 women who were selected as referents delivered in the hospital; of these, 62 women had antepartum stillbirths and were therefore excluded from the referent population and added to the case population. There were a total of 307 antepartum stillbirths in the hospital, giving an antepartum stillbirth rate of 13.6 per thousand births (Fig. 2).

When the demographic, social and obstetric characteristics of the case and referent populations were compared, the mean age for the case population was 25.7 years and for the referent women it was 23.7 years. Maternal age, maternal education, wealth index, antenatal care attendance and parity were different between the two groups (*p* < 0.001). Women in the case population were less educated, were from poorer families, had no antenatal care attendance and had more children (higher parity). In regards to obstetric complications, the case population had more previous stillbirths and a higher prevalence of antepartum hemorrhage and hypertensive disorder during pregnancy than the referent population (*p* < 0.001). The women in case population also had more small-for-gestational age babies than the referent population (*p* = 0.001) (Table 2).

**Table 2:** Background and social characteristics of case and referent populations

Variable	Referent	Antepartum Stillbirth	*p*-value^*^
	(N = 4505)	(N = 307)	
Maternal age in years			
Mean ± SD	23.7 ± 4.4	25.7 ± 5.7	
Median (IQR)	23.0 (20–26)	24.0 (21–29)	
Maternal age	*n (%)*	*n (%)*	
< 20	1232 (27.3)	57 (18.6)	*p* < 0.001
20–25	1965 (43.6)	114 (37.1)	
26–30	980 (21.8)	80 (26.1)	
> 30	328 (7.3)	56 (18.2)	
Maternal education			
Primary school (5 years) or less	1461 (32.4)	50 (16.3)	*p* < 0.001
Six years of schooling or more	3044 (67.6)	257 (83.7)	
Ethnicity			
Brahmin/Chhetri (Hill or Terai)	1743 (38.7)	114 (37.1)	
Relatively advantaged Janajatis	817 (18.1)	60 (19.5)	
Disadvantaged Janajatis	1299 (28.8)	100 (32.6)	
Non-Dalit (Terai)	373 (8.3)	16 (5.2)	
Dalit (Hill and Terai)	239 (5.3)	14 (4.6)	
Muslim	34 (0.8)	3 (1.0)	
Wealth Quintile			
Poorest	791 (19.1)	76 (40.6)	*p* < 0.001
Poorer	808 (19.5)	27 (14.4)	
Middle	865 (20.9)	22 (11.8)	
Richer	838 (20.2)	27 (14.4)	
Richest	846 (20.4)	35 (18.7)	
Antenatal Care Attendance			
At least one visit	3923 (87.1)	185 (60.3)	p < 0.001
No ANC	582 (12.9)	122 (39.7)	
Parity			
Primiparity	2432 (54.0)	137 (44.6)	p < 0.001
Multi-para (1–2)	1881 (41.8)	125 (40.7)	
Multi-para (3 or more)	192 (4.3)	45 (14.7)	
Previous Stillbirth			
No	4407 (97.8)	281 (91.5)	p < 0.001
Yes	98 (2.2)	26 (8.5)	
Antepartum hemorrhage during pregnancy			
No	4377 (97.2)	271 (88.3)	p < 0.001
Yes	128 (2.8)	36 (11.7)	
Hypertensive disorder during pregnancy	n (%)	n (%)	
No	4193 (93.1)	265 (86.3)	p < 0.001
Yes	312 (6.9)	42 (13.7)	
Medical problem during pregnancy			
No	4289 (95.2)	295 (96.1)	
Yes	216 (4.8)	12 (3.9)	
Sex of newborn			
Female	2115 (46.9)	136 (44.3)	
Male	2390 (53.1)	171 (55.7)	
Multiple birth			
No	4463 (99.1)	300 (97.7)	p < 0.023
Yes	42 (0.9)	7 (2.3)	
Small-for-gestational age			
Appropriate-for-gestational age	2811 (62.4)	161 (52.4)	p = 0.001
Small-for-gestational age	1694 (37.6)	146 (47.6)	

^*^p-value determined by *t*-test, Pearson’s chi-square analysis or Fisher’s exact test

Univariate logistic regression analysis showed that the odds of antepartum stillbirth increased by 10 % with each increasing year of maternal age (cOR 1.1, 95 % CI 1.07–1.1). The risk of antepartum stillbirth was 2.5 times higher in less educated woman compared to more educated (cOR 2.5, 95 % CI 1.8–3.4), and 2.5 times higher in the poorest women compared to the non-poor (cOR 2.5, 95 % CI 1.8–3.4). Women who had not attended any antenatal care visits had 4.5 times higher risk of stillbirth compared to those who had attended at least one antenatal care visit (cOR 4.5, 95 % CI 3.5–5.7). Similarly, the likelihood of antepartum stillbirth increased by 50 % if the women had previously been pregnant compared to those who were primiparous (cOR 1.5, 95 % CI 1.4–1.6). Women who had previous stillbirth had four times higher risk of antepartum stillbirth compared to those who did not (cOR 4.2, 95 % CI 2.7–6.5). The women with antepartum hemorrhage and/or hypertensive disorder during pregnancy had a 4.5 times increased risk of antepartum stillbirth (cOR 4.5, 95 % CI 3.1–6.7). Finally, the women who delivered small-for-gestational age babies had a 50 % higher likelihood for antepartum stillbirth than those who had appropriate weight-for-gestational age (cOR 1.5, 95 % CI 1.2–1.9) (Table 3).

**Table 3:** Univariate logistic regression analysis for likelihood of antepartum stillbirth

Variable	Crude Odds Ratio^a^ (cOR)	95 % CI
Maternal Age in years (linear)	1.1	1.07–1.1
Maternal education		
Six years of education or more	Ref	
Primary school (5 years) or less	2.5	1.8–3.4
Wealth index		
Non-poor	Ref	
Poor	2.5	1.8–3.4
Antenatal Care Attendance		
At least one visit	Ref	
No ANC	4.5	3.5–5.7
Parity		
Primi	Ref	
Multi-parity	1.5	1.4–1.6
Previous stillbirth		
No	Ref	
Yes	4.2	2.7–6.5
Antepartum hemorrhage in pregnancy		
No	Ref	
Yes	4.5	3.1–6.7
Hypertensive disorder during pregnancy		
No	Ref	
Yes	4.5	3.1–6.7
Small-for-gestational age		
Appropriate-for-gestational age	Ref	
Small-for-gestational age	1.5	1.2–1.9

^a^Univariate logistic regression analysis to determine the likelihood of antepartum stillbirth

Multivariate logistic regression analysis was conducted to adjust for the interaction of exposure variables with one other. In this model, the risk of antepartum stillbirth was still increased among women with increasing age, who had no education, belonged to the poorest families, had a higher parity, who did not go for antenatal care checkups, had a previous stillbirth, had antepartum hemorrhage or hypertensive disorder during pregnancy, and/or who had small-for-gestational age babies (Table 4).

**Table 4:** Multivariate logistic regression analysis for likelihood of antepartum stillbirth

Variables	Adjusted Odds Ratio^a^	95 % CI
	(aOR)	
Maternal age (in years)	1.0	1.0–1.1
Primary school (5 years) education or less	2.4	1.7–3.2
Parity	1.2	1.0–1.3
Previous stillbirth	2.6	1.6–4.4
No antenatal care attendance	4.2	3.2–5.4
Poorest family	1.3	1.0–1.8
Antepartum hemorrhage	3.7	2.4–5.7
Hypertensive disorder during pregnancy	2.1	1.5–3.1
Small-for-gestational age	1.5	1.2–2.0

^a^aOR has been adjusted to maternal age, educational status of mother, parity, previous stillbirth, antenatal care, socio-economic status, antepartum hemorrhage, hypertensive disorder during pregnancy and small for gestation age in the table which had cOR > 1

## Discussion

Through this study we found that the risk of antepartum stillbirth was higher among women with less than five years of education, who belonged to the poorest family, who were older, had higher parity and who did not attend any antenatal care visits. Similarly, the risk of antepartum stillbirth was also increased for women who had a previous stillbirth, antepartum hemorrhage or hypertensive disorder during pregnancy, or small-for-gestational age babies in a tertiary hospital setting in Nepal. Similar to our results, studies in developed countries have identified several modifiable risk factors for antepartum stillbirth such as lack of antenatal care, antepartum hemorrhage, hypertensive disorder during pregnancy, and small-for-gestational age babies, however, the socio-economic and health service settings were different [4, 7, 20]. A study conducted in India has also identified lack of antenatal care as a modifiable risk factor for stillbirth [21].

There are several limitations to this study. First, some of the potential risk factors for antepartum stillbirth, such as placental insufficiency or genetic disorders, could not be assessed due to the lack of placental examination, both grossly and microscopically, and gene analysis. Similarly, not all women, even those who had antenatal care, had screening for medical and/or obstetric complications during pregnancy, so there could be under-reporting of these conditions. Secondly, this is a case-control study, which can only demonstrate an association between the various risk factors and antepartum stillbirth, but cannot determine the causal relationship. Third, there may have been some potential bias within this study, such as failure of health workers to correctly assess maternal medical conditions during the clinical examination at admission. Fourth, the population-based references for determining birth weight according to gestational age and sex were not available for the Nepali setting, so US population-based references were used. Finally, since this was a hospital-based study, the background characteristics of women having antepartum stillbirths might be different at the population level.

There are possible explanations for the associations seen between some of these risk factors and antepartum stillbirth. For example, women with hypertensive disorder during pregnancy are more likely to have placental compromise, and thus a higher risk for fetal death. Additionally, women with increasing maternal age are more likely to have chronic hypertension and placental pathologies [22].

Studies have shown that antenatal care given by a skilled healthcare provider is a cost-effective intervention [23–25]. These visits provide a screening opportunity for certain risk factors that are shown to be associated with antepartum hemorrhage, certain medical conditions, infection or hypertensive disorder [23–25]. If risks are detected, healthcare providers have the opportunity to immediately manage or treat specific conditions, or to establish a future care plan. Additionally, they can provide counseling to mothers and families, all of which can help to prevent antepartum stillbirth.

In Nepal, the government of Nepal provides free antenatal care check-ups through its public health facilities, and has a standard protocol for antenatal check-ups [26]. Nevertheless, only two-thirds of women in the country go for antenatal check-ups from a skilled healthcare provider, and only 61 % of women who do go receive adequate antenatal check-ups (e.g. blood pressure examination, urine and blood tests) [10]. Moreover, large disparities exist in the access to antenatal care by socioeconomic status, with women from the poorest quintile and those with the least education having the lowest access to care in Nepal [27].

## Conclusions

Our study from Nepal, the first in the country, investigating the risk factors for antepartum stillbirth showed that antepartum stillbirth was associated with adverse social, demographic and obstetric conditions in the mothers. Among the several risk factors we identified, antenatal care from a skilled provider can reduce the risk of antepartum stillbirth, and increasing access to and quality of antenatal care will be of utmost important to screen for maternal morbidity, as well as fetal growth. Improvements in access to and quality of antenatal care should be coupled with changing awareness on the importance of antenatal care and increasing the knowledge of the risk factors for adverse birth outcomes. With the government of Nepal committing to the Global Every Newborn Action Plan’s goal to reduce the number of stillbirths by more than two-thirds of the 2015 level by 2035, more investment and studies are required to identify strategies to increase the access to and quality of antenatal care among poor and uneducated women.
